# Mechanisms of the effect of gut microbes on depression through the microbiota-gut-brain axis

**DOI:** 10.3389/fnut.2025.1634548

**Published:** 2025-08-06

**Authors:** Xiangyan Zhou, Sixing Wang, Xiaohui Wang, Xinran Chen, Pei Zhou, Kai Ma, Peng Zhang

**Affiliations:** ^1^Department of Gastrointestinal Surgery, Union Hospital, Tongji Medical College, Huazhong University of Science and Technology, Wuhan, China; ^2^First Clinical Department, Tongji Medical College, Huazhong University of Science and Technology, Wuhan, China; ^3^Second Clinical Department, Tongji Medical College, Huazhong University of Science and Technology, Wuhan, China; ^4^The Center for Biomedical Research, Department of Respiratory and Critical Care Medicine, NHC Key Laboratory of Respiratory Diseases, Tongji Hospital, Tongji Medical College, Huazhong University of Science and Technology, Wuhan, China

**Keywords:** gut microbiota, MGB axis, depression, metabolism, neurotransmitters, neuroinflammation

## Abstract

Depression is a significant public health issue which exerts profound psychological and social impacts on both individuals and society. However, existing therapeutic strategies often exhibit limited efficacy. Accumulating evidence underscores the vital role of gut microbiota in the pathophysiology of depression through the microbiota-gut-brain (MGB) axis. This involves multiple mechanisms, including short-chain fatty acid (SCFA) metabolism, communication via the vagal nerve, regulation of the hypothalamic-pituitary-adrenal (HPA) axis, and immune-inflammatory interactions. This review provides a comprehensive review of the mechanisms through which gut microbiota influences depression via the MGB axis. It synthesizes recent achievements in this field and evaluates the potential of microbiome-targeted therapies for depression treatment. Furthermore, it outlines future research directions to establish a theoretical framework for novel therapeutic approaches and to foster the development of this area.

## 1 Introduction

Depression is the most prevalent and debilitating mental disorder, ranking as the third leading cause of global disease burden (1). The etiology of depression is multifactorial. For instance, the COVID-19 pandemic can increase the global incidence of depression by 25% (2). Additionally, adolescents with >3 h of daily screen time have a 34% increased risk of developing depression (3). Nowadays, the primary therapy for depression relies heavily on antidepressant medicines, with selective 5-hydroxytryptamine (5-HT) reuptake inhibitors being the most commonly used. However, approximately 30% of patients exhibit treatment resistance or achieve suboptimal therapeutic outcomes (4). Recently, the role of gut microbiota in depression has garnered increasing attention. Emerging studies have uncovered novel pathways through which gut bacteria can influence depression via the MGB axis. The results of this research lay the foundation for the development of novel therapeutic strategies, thereby overcoming the shortcomings of traditional antidepressant therapies.

The human gastrointestinal system hosts a symbiotic microbiota community, which consists of bacteria, fungi, and viruses. Its biomass exceeds that of human cells by over 1.5 times (5–7). The gut bacteria, which make up more than 90% of the microbiota, are primarily composed of *Firmicutes*, *Bacteroidetes*, and *Actinobacteria*, though it varies significantly among individuals (8). The gut microbiota exerts significant effects on human health through various mechanisms: (1) maintenance of intestinal barrier integrity through the production of short-chain fatty acids (SCFAs), B/K vitamins, amino acids, and bile acids (BAs) (9–12); (2) eliminating invasive intestinal pathogens and reducing their colonization (13, 14); (3) regulating inflammatory cytokines and promoting immune system maturation (15). Patients with depression often exhibit reduced gut microbiota diversity and dysbiosis, while restoring the microbiome is linked to improvements in depressive symptoms (16, 17). Substantial evidence highlights the essential role of gut microbiota in influencing depression via the MGB axis.

The MGB axis refers to the bidirectional communication system linking the brain and the gut. It forms a homeostatic network that encompasses neurological, endocrine, and immunological pathways (18). Various mechanisms are implicated, including SCFA metabolism, the monoamine neurotransmitter system, the vagal nerve pathway, the HPA axis, and immune signaling. However, the specific mechanisms underlying these processes are still not fully understood. This review aims to explore how the gut microbiota influences depression via the MGB axis, providing valuable insights for depression research.

Relevant studies were searched in the PubMed, Web of Science, and CNKI databases, including original research articles, observational studies, and reviews. The search strategy terms included gut microbiota, MGB axis, major depressive disorder and depression. The search was restricted to articles published in English and included studies on all species without restrictions on publication date. From an initial pool of 412 publications, we applied rigorous selection criteria focusing on: Mechanistic studies of gut-brain interactions in depression, clinical/preclinical intervention studies with robust methodologies. High-quality reviews and meta-analyses. After thorough screening of abstracts and full texts, 206 studies were ultimately selected based on their scientific quality, relevance to our review objectives, and publication impact.

## 2 Gut microbiota and depression

**Figure 1:**
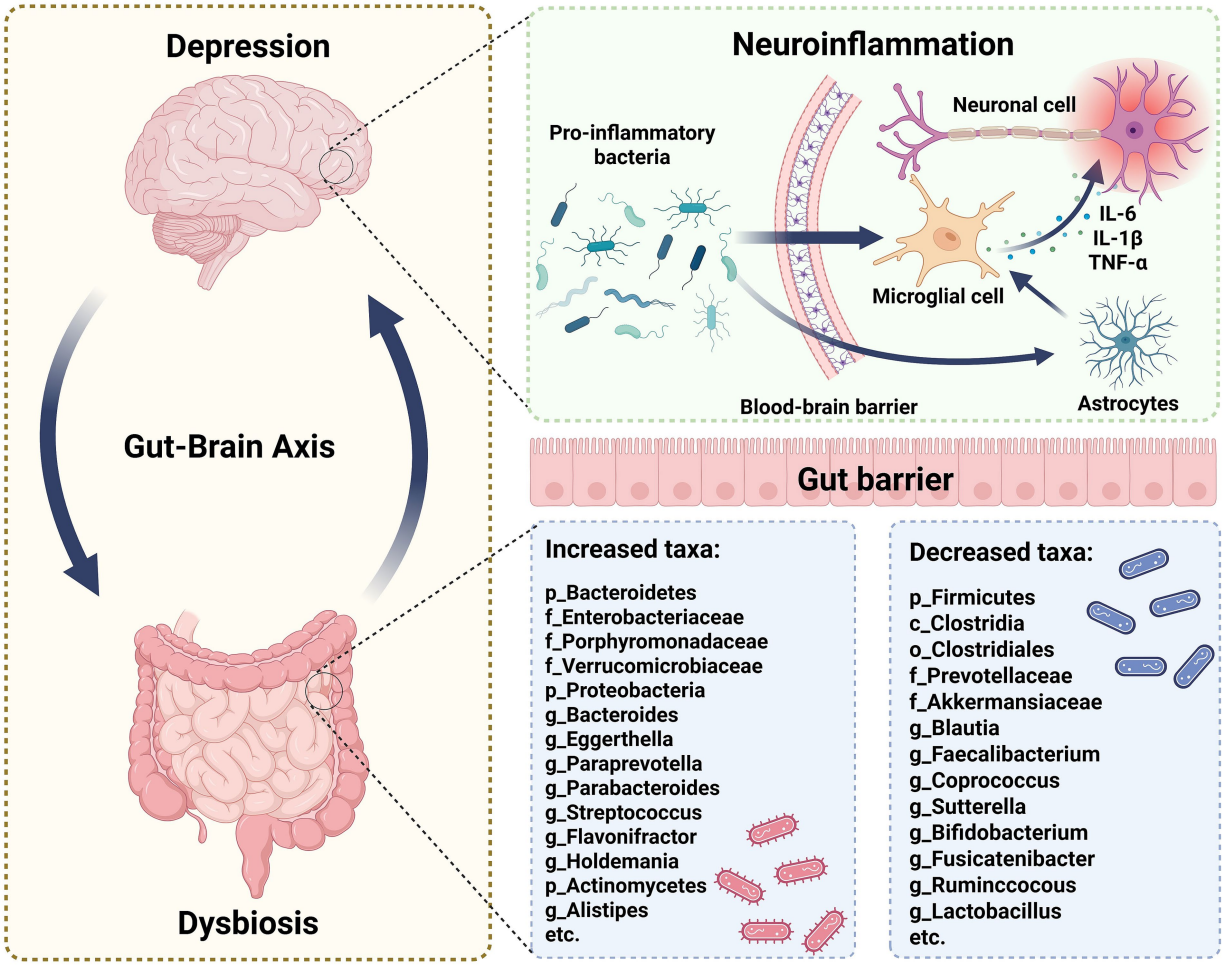
The specific alterations in gut microbiota associated with depression. Depression and gut microbiota dysbiosis share a robust bidirectional relationship. Depression may exacerbate gut dysbiosis, and conversely, dysbiosis can negatively affect mental health. Specifically, certain pro-inflammatory bacteria could penetrate the blood-brain barrier, activating microglia. This activation increases the level of cytokines, such as IL-6, IL-1β, and TNF-α, which can induce neuroinflammation. Moreover, in the gastrointestinal tract of individuals with depression, significant changes in microbiota composition occur, characterized by increased relative abundance of certain microbes and decreased beneficial microbes. These gut microbiota alterations may influence depression development through gut-brain axis mechanisms.

Recent studies have increasingly demonstrated a significant association between alterations in gut microbiota and depression (19). Individuals with depression exhibit marked gut microbiota dysbiosis (Figure 1) (20–22), characterized by elevated levels of *Enterobacteriaceae*, *Alistipes*, *Bacteroidetes*, *Proteobacteria*, and *Actinobacteria*, while *Faecalibacterium* and *Firmicutes* are notably reduced (23). These gut microbiome changes are evident across different age groups in individuals with depression (24–27).

With advancements in macro-genomics and metabolomics, more research has focused on precisely characterizing the gut microbiota alterations related to depression. For instance, the genera *Alistipes*, *Anaerostipes*, and *Dialister* are significantly reduced in individuals with active depression, while *Haemophilus* is increased in those with mild depression (28, 29). Bacteroidetes levels are markedly elevated in people with mild depression, accompanied by reductions in *Faecalibacterium* and *Escherichia*; however, severe depression patients show increased *Bacteroidetes* but decreased *Ruminococcus* and *Eubacterium* (29). The composition of gut microbiota not only varies across different depressive states but also exhibits distinct changes across different depressive species. For instance, a decrease in the *Alistipes* has been observed in mouse depressive models (30). Therefore, how to accurately and precisely determine the composition of gut microbiota under depressive states remains a critical issue that needs to be addressed.

Some evidence suggests that gut microbiota can directly translocate to the brain parenchyma when the intestinal barrier and the blood–brain barrier (BBB) are compromised, in addition to indirectly influencing depression via the MGB axis (16, 31). This translocation of gut microbiota into the brain may lead to distinct inflammatory changes in different brain regions over time. Research indicates that during the acute phase of depression models, pro-inflammatory gut microbiota primarily infiltrate the brain and induce inflammation, while in the chronic phase, microbiota that penetrate the brain mainly activate neurodegenerative pathways (31). However, it remains unclear whether similar time-dependent changes in gut microbiota occur in the brains of depressed patients. If such changes exist, they could provide a basis for targeted treatments at different stages of depression. Moreover, the effects of gut microbiota invasion vary across different brain regions (31). Given that different depression symptoms may be linked to changes in specific brain regions, future studies could utilize spatial transcriptomics to analyze inflammatory differences across brain regions in mouse models of depression and correlate these with symptomatic changes. This approach could help determine whether gut microbiota induce different depressive symptoms by mediating inflammatory responses in distinct brain regions. The finding that gut microbiota can directly enter the brain and trigger neuroinflammation is interesting and novel, providing a new mechanism and potential new therapies for the onset of depression. However, the mechanism by which gut microbiota can directly enter the brain through damaged intestinal barriers and blood–brain barriers only exists under the specific condition of “intracortical microelectrode implantation,” which is a very special case. Whether this mechanism exists in general patients remains to be explored. If it does exist, it may be possible to alleviate depression by repairing the intestinal barriers and blood–brain barriers. Besides, current studies are limited to animals, with a lack of research in humans. In the future, more research can be conducted in this area.

## 3 Gut microbiota affects depression through the MGB axis

**Figure 2:**
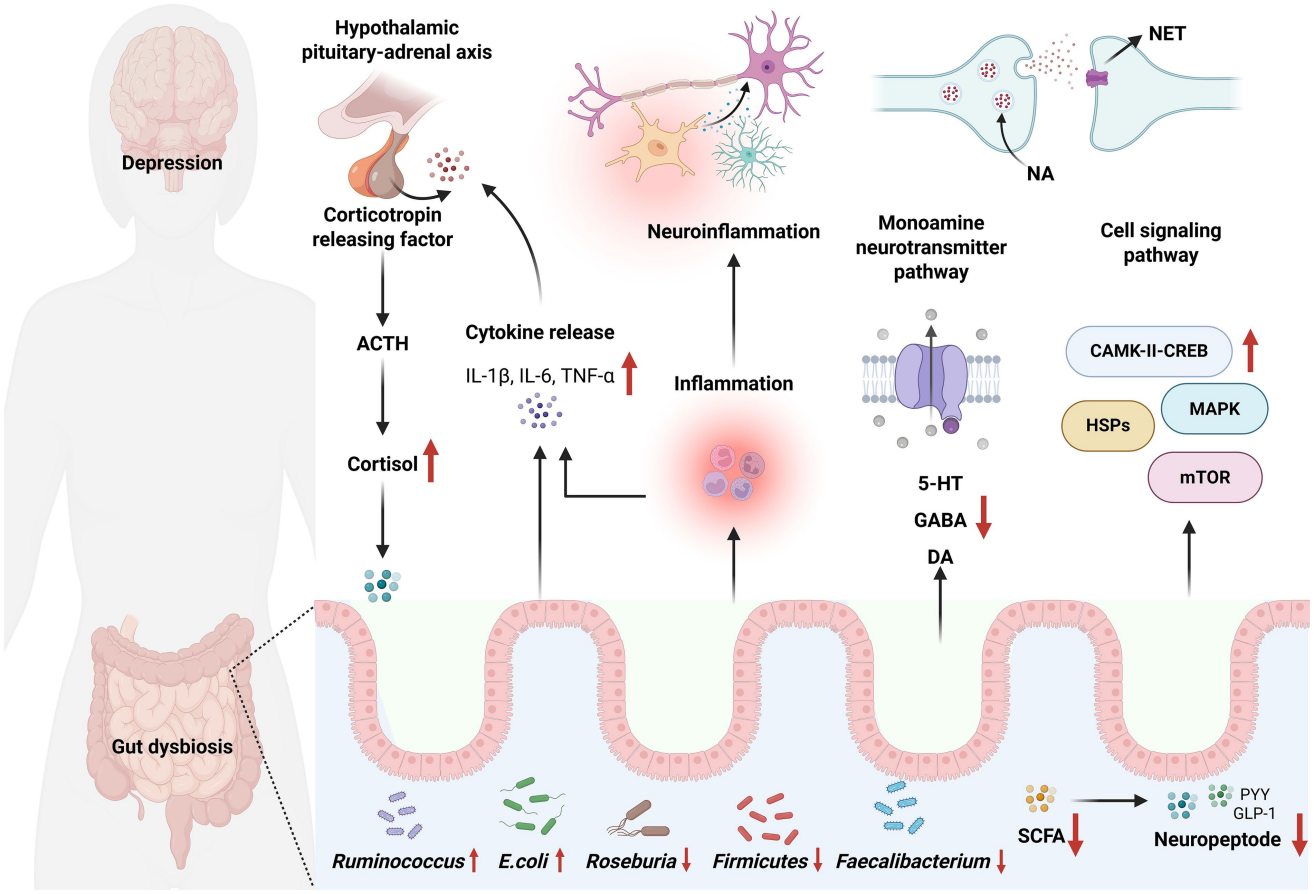
Gut microbiota affects depression through the MGB axis. The HPA axis regulates cortisol levels via corticotropin-releasing factor and adrenocorticotropic hormone. Concurrently, the release of IL-1β, IL-6, and TNF-α can induce neuroinflammation and modulate the HPA axis. Moreover, gut dysbiosis can impact monoamine neurotransmitter pathways and cell signaling pathways, including those involving 5-HT, γ-aminobutyric acid (GABA), dopamine (DA), and mammalian target of rapamycin (mTOR). Alterations in gut microbiota, particularly the reduction of beneficial bacteria and the increase of harmful bacteria, may influence neuropeptide expression. Additional mechanisms by which gut microbiota influence depression via MGB axis are listed in Table 1.

MGB axis is important in the etiology of depression (Figure 2). The gut microbiota influences the onset of depression through many pathways mediated by the MGB axis, including metabolism, neurotransmitters, neurological pathways, and endocrine systems. Figure 2 illustrates a prevalent MGB axis pathway associated with depression.

**Table 1:** Mechanisms of the effect of gut microbes on depression through the microbiota-gut-brain axis.

Pathway	Represent	Mechanisms	Intervention	References
SCFA metabolism	Acetic acid, propionic acid, butyric acid, valeric acid, isovaleric acid, hexanoic acid, isocaproic acid	Regulating intestinal pH and maintaining intestinal microbiota balance; enhancing intestinal barrier function; alleviating neuroinflammation; modulating of neural activity; enhancing neuroplasticity; stimulating neuropeptide release; regulating of 5-HT secretion	Supplementation with butyrate, probiotics (*Bifidobacterium*, *Lactobacillus*), or synbiotics (containing *P. prausnitzii*, oligofructose, and oligogalactose) increased SCFAs levels Mediterranean-DASH intervention for neurodegenerative delay diets and high-fiber diets increase beneficial SCFAs-producing bacteria Supplementation with *Saccharomyces cerevisiae* increases SCFAs levels by increasing β-glucan	(34, 36–46, 49, 50, 126, 205)
Bile acid metabolism	Chenodeoxycholic acid, glycolithocholic acid, taurolithocholic acid, lithocholic acid-3-sulfate	Regulating lipid metabolism; regulating 5-HT and GABA signaling via TGR5 and FXR receptors; inhibiting NLRP3 inflammatory vesicle activity; regulating GLP-1 secretion; regulating oil-previous ethanolamide levels	Probiotics (*Blautia*, *Eubacterium*) restore secondary bile acid metabolism TGR5 agonists improve depressive-like behavior	(55–61, 64)
Tryptophan metabolism	5-HT Quinolinic acid Kynurenine melatonin	Decreasing levels lead to mood disorders Suppressing neuroprotective effects Promoting neuroinflammation Conversion to neuroprotective or neurotoxic metabolites Regulating the sleep–wake cycle	The combination of *A. muciniphila* and enteric lactate restores tryptophan metabolic balance Supplementation with probiotics such as *Bifidobacterium infantis* promotes canine uridine conversion	(69, 72–76, 78–80)
Other metabolism	Lactic acid LPS Glutamatergic neurotransmitter Sphingolipid metabolites TMAO	Preventing and reversing depression Promoting neuroinflammation Reflecting depression severity Promoting intestinal microbiota balance and improving the intestinal barrier Promoting the secretion of pro-inflammatory factors that lead to neurotransmitter death	Escitalopram improves depression by upregulating sphingolipid metabolism; fingolimod (sphingosine-1-phosphate receptor modulator) improves depression; milk sphingomyelin improves gut microbiota balance; phosphatidylcholine (PC) and sphingomyelin (SM) regulate tryptophan metabolism, bile acid metabolism, and vitamin digestion and absorption	(81–91, 93)
Monoamine neurotransmitter	NE 5-HT DA	Abnormal metabolism leading to disruption of NE signaling system Inhibiting inflammatory response; affecting GI and CNS function via vagus nerve Elevated homocysteine levels damage dopaminergic neurons; metabolic disorders lead to decreased signaling and directly affect mood	α2-adrenergic receptor antagonists promote noradrenergic neurotransmission *L. zhachilii HBUAS52074T* increases serum 5-HT concentration Folic acid supplementation promotes homocysteine metabolism; DA agonists improve depression associated with Parkinson’s disease	(83, 97, 98, 100–106, 109, 110, 112, 113, 116)
Amino acid neurotransmitter	GABA	Binding to enteric neurons and vagal receptors to regulate intestinal activity; blocking ion flow at the postsynaptic membrane to reduce neuronal excitability	*Lactobacillus rhamnosus* intervention upregulates GABA levels and activates the brain-derived neurotrophic factor-protomyosin kinase B signaling pathway; *Lactobacillus rhamnosus* intervention alters brain GABARs expression; acetate can participate in the GABA glial cell cycle	(120–123)
Neuropeptide neurotransmitter	GLP-1, peptide YY	Regulating intestinal function; influence on gut microbiota composition; transvagal signaling and inhibition of CRH neuronal activity; promoting monoamine neurotransmitter release; enhancing neuroplasticity and repairing neuronal damage	GLP-1 receptor agonists inhibit CRH synthesis	(83, 127)
Vagus nervous system	Acetylcholine	Increasing monoamine neurotransmission at synapses; acetylcholine binds to cholinergic receptors on immune cells and inhibits the release of inflammatory factors	Vagus nerve stimulation; percutaneous stimulation of the vagus nerve	(132–137, 139–141)
Enteric nervous system	Enteric glial cell Enteroendocrine cells	EGC inflammatory subpopulation triggers intestinal inflammation via the CSF1/TNF-α pathway; EGC is involved in maintaining intestinal epithelial barrier function ECC secretes neuropeptides, cholecystokinin, and regulates 5-HT and kynurenine metabolism	Supplementation of SCFA to maintain ENS function	(142, 144, 145)
Cell signaling pathway	MAPK/CREB pathway Endogenous cannabinoid system mTOR pathway Heat shock protein pathway	Downstream CAMK II-CREB cascade response mediates gut microbiota-brain-gut axis messaging Binding hippocampus-specific receptors and modulating memory-emotion encoding Influencing synaptic plasticity and neural activity Regulating BDNF expression and exerting neuroprotective effects		(83, 151–154)
Immunity	LPS, IL-10, IL-22, microglia, T cells, B cells, NK cells	Causing imbalance in canine uridine metabolism; impairing neuroplasticity and induces neuroinflammation; inhibiting hippocampal development; exacerbating disorders of neuronal energy metabolism	*Clostridium difficile* provides growth factors and regulates intestinal immune balance; supplementation with *Lactobacillus rhamnosus* improves anti-inflammatory factors; metabolites such as SCFAs to modulate Treg/Th17 balance; exogenous IL-12 intervention; supplementation with *Saccharomyces cerevisiae* inhibits IL-6 production by increasing β-glucan	(157–162, 164, 166, 169, 172, 174)
Endocrine	Cortisol, CRH, ACTH	Impairing immune function; interfering with gastrointestinal function; increasing intestinal permeability and inducing bacterial translocation	Supplementation of *Pichia pastoris KM71H* and *Saccharomyces boulardii* reduces corticosterone levels in the mouse hippocampus and inhibits HPA axis overactivation	(159, 161)

### 3.1 Metabolism

#### 3.1.1 SCFAs

SCFAs, including acetic, propionic, and butyric acids along with their salts and esters, are key metabolites produced by gut microbiota (32). SCFA production is influenced by diet and gut microbiota composition, with *Bacteroidetes*, *Firmicutes*, and *Actinobacteria* being notable producers (33).

SCFAs are often deficient in individuals with depression, and their supplementation can improve depressive symptoms (34, 35). The mechanisms involved include: (1) Regulating intestinal pH: SCFAs, mainly present as anions in the gut, lower intestinal pH. This favors the growth of beneficial bacteria like Lactobacillus (optimal pH 3.0–4.5) over pathogenic bacteria such as *Salmonella* and *Staphylococcus* (optimal pH 6.0–7.0) (36). (2) Improving intestinal barrier function: SCFAs have demonstrated the potential to mitigate chronic stress-induced elevations in intestinal barrier permeability and facilitate the development of tight junctions within the intestinal barrier (37, 38). Moreover, SCFAs can maintain the integrity of the intestinal mucosa by regulating the transcription and expression of related genes such as specific protein 1 (39). Low concentrations of butyrate enhance barrier function, whereas high concentrations can be detrimental (40). (3) Exerting anti-inflammatory effects: SCFAs bind to the free fatty acid receptor 3, inhibiting microglia M1 polarization, reducing pro-inflammatory cytokines such as IL-6, TNF-α, and increasing anti-inflammatory cytokines such as IL-10 to mitigate neuroinflammation (41, 42). (4) Stimulating of neuropeptide release: SCFAs enhance the release of peptides while reducing hunger hormone secretion (39). (5) Epigenetic mechanisms: SCFAs inhibit histone deacetylase (HDAC), promoting brain-derived neurotrophic factor (BDNF) gene transcription and upregulating hippocampal BDNF expression to enhance neuroplasticity (43). Butyrate also reduces DNA methylation by activating methylcytosine dioxygenase 1, rescuing gene silencing and restoring BDNF expression (44). (6) Regulating neurotransmitters: SCFAs can stimulate 5-HT secretion, thereby influencing mood (45). (7) Crossing the BBB: SCFAs can traverse the BBB directly, modulate neurotransmission, and influence neuronal excitability (34).

Beyond acetic, propionic, and butyric acids, SCFAs also include valeric, isovaleric, hexanoic, and isocaproic acids. Though less studied, these SCFAs may also play roles in depression. For instance, valproate (a valeric acid derivative) affects neuronal function, reduces neuroinflammation, alters gut microbiota, and maintains intestinal barrier integrity (46, 47). Valeric acid diminishes TNF-α levels and regulates the immunological response (48). Isovaleric acid can cross the BBB and disrupt neurotransmitter release, potentially worsening depression (49). Caproic acid is recognized for its roles in metabolic regulation, antibacterial activity, and anti-inflammatory actions (50). The production and function of SCFAs in depression remain incompletely understood, particularly regarding dose-dependent effects as exemplified by butyrate’s dual roles at different concentrations. The transport dynamics of various SCFAs across the blood–brain barrier require further elucidation, while data on minor SCFAs in human depression remain limited. Future studies should establish optimal therapeutic windows for SCFA interventions, develop targeted delivery systems to enhance brain bioavailability, and investigate potential synergistic effects of SCFA combinations as novel antidepressant strategies. The differential effects of various SCFA formulations (salts vs. esters) and their long-term safety profiles also warrant systematic evaluation in clinical populations.

#### 3.1.2 BAs

Gut microbiota dysregulation can influence depression by altering BA concentrations (51). Studies have shown that Blautia and Eubacterium, which are crucial for converting secondary bile acids, are reduced in individuals with depression (52, 53). Depressed patients exhibit significantly lower levels of chenodeoxycholic acid, lithocholylglycine, taurolithocholic acid, and lithocholic acid-3-sulfate, while levels of 23-demethoxycholic acid are significantly higher compared to healthy controls (54). BAs affect lipid metabolism and mediate physiological effects, including immune regulation, by binding to the farnesoid X receptor (FXR) and Takeda G-protein-coupled receptor 5 (TGR5). Mice lacking FXR show reduced depressive behavior, whereas those deficient in TGR5 exhibit increased depression-like behavior (55, 56). Thus, BA metabolism regulated by gut microbiota may influence the MGB axis via TGR5 and FXR, thereby affecting depression development. The specific mechanisms are as follows: (1) Affecting neurotransmitter levels: TGR5 deficiency significantly reduces 5-HT levels in serum and decreases 5-HT1A receptor expression in the hippocampal region of mice, indicating that TGR5 is vital in regulating the 5-HT nervous system (57). Notably, TGR5 expression is reduced in GABA neurons in the lateral hypothalamic area (LHA) of depression model mice. Mechanistically, TGR5 bidirectionally modulates GABA neuronal excitability in the LHA via extracellular regulation of protein kinase-dependent Kv4.2 potassium ion channels. Its antidepressant-like effect stems from the inhibition of GABAergic neuronal inhibitory output in the LHA by TGR5 (58). FXR deficiency reduces the level of the GABA synthase glutamic acid decarboxylase 65 in the hippocampus and upregulates the level of the GABA transporter protein GABA transporter 1, leading to abnormal GABA metabolism. Notably, GABA, 5-hydroxyindole inhibitory acid, and GABA/glutamic acid ratios are elevated in FXR-deficient mice, suggesting that BAs are involved in the pathogenesis of depression through the modulation of neurotransmitter homeostasis (56). (2) Modulating the immune response: FXR and TGR5 can inhibit pro-inflammatory gene expression by regulating endogenous inflammatory vesicles (59). Chenodeoxycholic acid treatment can downregulate NOD-like receptor thermal protein domain associated protein 3 (NLRP3) inflammatory vesicle activity via FXR (60). (3) Regulating glucagon-like peptide-1 (GLP-1) secretion: BAs are capable of indirectly enhancing GLP-1 signaling through the activation of TGR5 (61). GLP-1 receptor agonists improve depression-like behavior in LPS-treated and obese rodents, possibly by affecting DA synthesis and metabolism or promoting mitochondrial autophagy to inhibit microglial cell focal death (62, 63). (4) Modulating oil-precursor ethanolamide (OEA) levels: Although the exact relationship between BAs and OEA levels has not been fully clarified, BAs increase OEA levels in the jejunum of mice (64). OEA treatment increases Norepinephrine (NE) and 5-HT in the brain and activates peroxisome proliferator-activated receptor α, which, in turn, activates hindbrain neurons and substantia nigra striatal dopaminergic neurons, thereby improving depressive-like behavior in mice (65–67). While bile acids (BAs) show promise in modulating depression via TGR5/FXR pathways, key gaps remain in understanding their precise neuroactive mechanisms, human-specific effects, and clinical applicability. Future work should focus on: human BA metabolomics to identify depression biomarkers, developing CNS-targeted BA receptor modulators, and exploring dietary interventions to optimize BA profiles. Integrating BA research with other gut-brain signals may yield novel therapeutic strategies.

#### 3.1.3 Tryptophan

**Figure 3:**
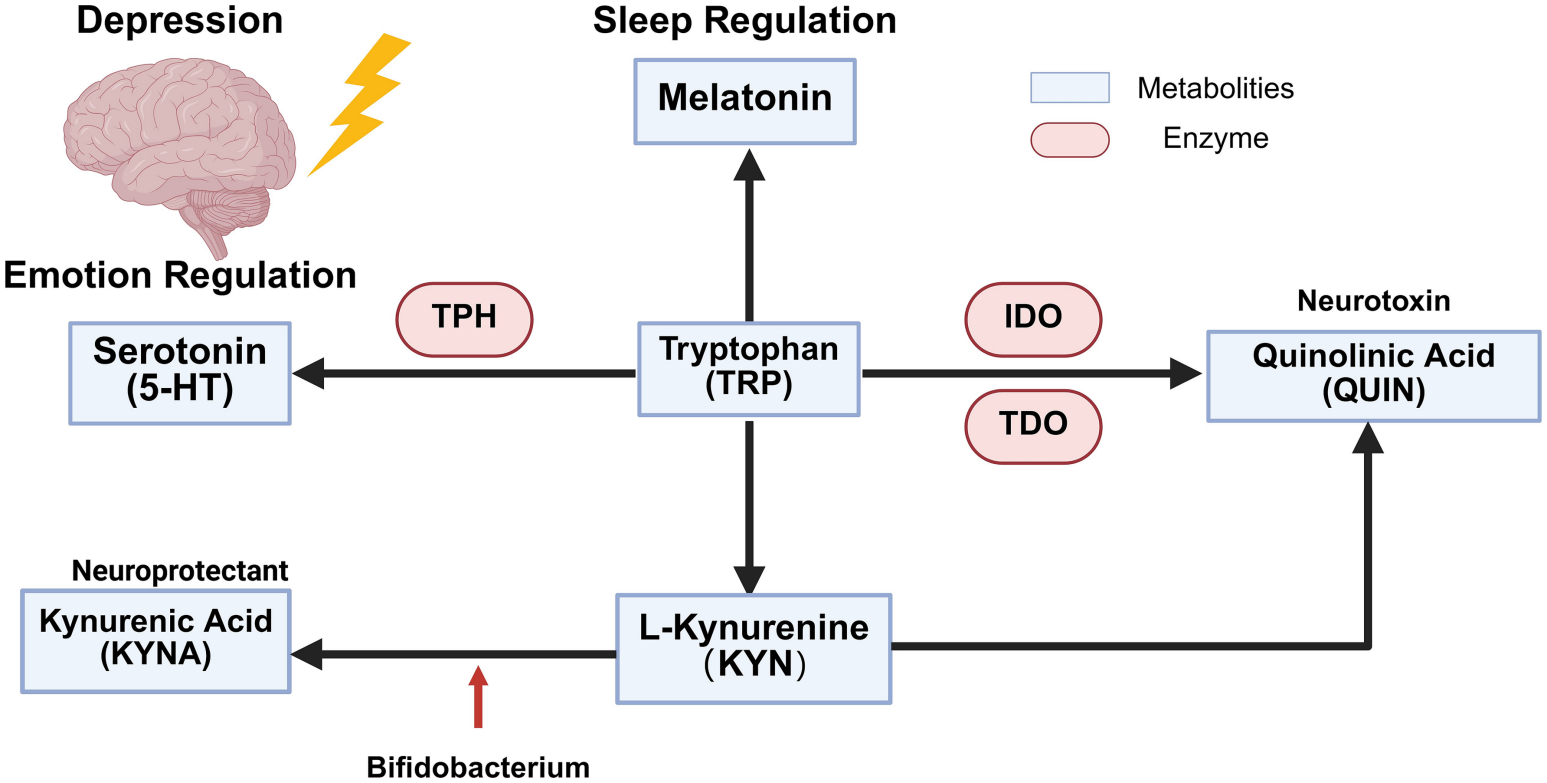
Tryptophan metabolism in depressed patients. Tryptophan is metabolized by tryptophan hydroxylase into 5-HT, a neurotransmitter involved in mood regulation, and into melatonin, which plays a role in sleep regulation. Additionally, enzymes such as indoleamine-2,3-dioxygenase and tryptophan 2,3-dioxygenase convert tryptophan into the neurotoxic metabolite QUIN. Tryptophan can also be converted into KYN, which is subsequently transformed into KYNA by bifidobacteria, exerting neuroprotective effects.

Tryptophan, a vital amino acid, has metabolic pathways that are closely linked to depression development (68) (Figure 3). It is metabolized through four main pathways: (1) Tryptophan is converted to 5-HT (69). In the human body, 5-HT is distributed unevenly, with approximately 10% in the central nervous system and 90% in the gastrointestinal tract. The BBB prevents gastrointestinal 5-HT from entering the brain, limiting its role as a neurotransmitter there (70). Individuals with depression often have lower serum 5-HT levels (71). 5-HT production is highly dependent on tryptophan availability and tryptophan hydroxylase activity. Low plasma tryptophan levels are associated with impaired immune function, suggesting that such individuals may have inadequate 5-HT synthesis, making them more prone to depression-like symptoms (72). Additionally, the combination of *Akkermansia muciniphila* and intestinal lactate has been shown to restore tryptophan metabolism balance in people with anxiety disorders and enhance 5-HT effects, thus alleviating anxiety (73). (2) Quinolinic acid pathway: Tryptophan is enzymatically converted to quinolinic acid (QUIN) by tryptophan-2,3-dioxygenase or tryptophan-2,3-monooxygenase. This pathway reduces the conversion of tryptophan to 5-HT, diminishing its neuroprotective effects. Moreover, QUIN and its derivatives may promote neuroinflammation and depressive states (74). Clinically, a combined antidepressant strategy using indoleamine 2,3-dioxygenase 1 inhibitors and probiotics has been proposed and shown to be effective (75). (3) Kynurenine pathway: Over 95% of peripheral tryptophan is oxidized to kynurenine to eliminate excess tryptophan (76). Kynurenine can be further metabolized into kynurenic acid (KYNA), a neuroprotective agent, and QUIN, a neurotoxin (77). Evidence suggests that probiotics like *Bifidobacterium infantis* can promote the conversion of kynurenine to KYNA, thereby reducing stress-induced depression (78, 79). (4) Melatonin, a significant tryptophan metabolite primarily produced by the pineal gland, is crucial for regulating the sleep–wake cycle. Sleep disturbances in individuals with depression may be related to abnormal melatonin metabolism (80). Current understanding of tryptophan metabolism in depression lacks comprehensive human data on pathway crosstalk and individual variations. Future research should prioritize: (1) clinical validation of microbiota interventions (e.g., *Akkermansia*, *Bifidobacterium*) for tryptophan pathway modulation, (2) development of dual-acting therapies targeting both 5-HT synthesis and neuroinflammation (e.g., IDO inhibitors with probiotics), and (3) personalized approaches based on tryptophan metabolic profiling to optimize treatment outcomes.

#### 3.1.4 Other metabolic pathways

The gut microbiota can influence the development of depression through various metabolic pathways, such as those involving lactate, sphingolipids, and trimethylamine oxide (TMAO). Lactate has been demonstrated to inhibit and reverse depression (81). Lipopolysaccharides (LPS) produced by gut microbiota can exacerbate the inflammatory response associated with depression (82). Imbalances in gut microbiota can affect protein phosphorylation pathways in the hippocampus, contributing to the onset of depression (83). Additionally, disturbances in carbon and amino acid metabolism have been observed in the hippocampus of rats with depression (84). Elevated glutamatergic neurotransmission is significantly linked to depression, as evidenced by increased glutamate levels in serum, plasma, and brain tissue of individuals with depression. Moreover, plasma glutamate levels have been identified as indicators of depression severity (85–87).

Recent studies indicate that the stress-induced depression model in Wistar rats is associated with alterations in sphingolipid metabolism of phosphatidylcholine and sphingomyelin. Furthermore, the application of milk sphingomyelin, as opposed to egg sphingomyelin, fosters intestinal microbiota homeostasis and enhances the intestinal barrier in mice, potentially ameliorating depression (88–90). Escitalopram was found to enhance depressive symptoms by upregulating critical molecules involved in sphingolipid metabolism (sphingomyelin, sphingosine-1-phosphate). For the first time, an aberration in sphingolipid metabolism was linked to dysbiosis and neuroinflammation, providing a new viewpoint on the connection between sphingolipid metabolism and depression, as well as the MGB axis (91). It has been observed that serum TMAO levels are elevated in methamphetamine (METH)-exposed mice. Increased TMAO levels are associated with higher levels of pro-inflammatory factors, neuronal cell apoptosis, and depression-like behavior, with serum TMAO levels positively correlating with depressive symptoms (92). However, research on TMAO and depression remains limited. The mechanism by which intestinal bacterial metabolites cross the intestinal barrier and the BBB to induce neuroinflammation is still not fully understood. The newly established iPSC model has elucidated how butyric acid can traverse the BBB via the monocarboxylic acid transporter protein (MCT) to stimulate BDNF expression in the hippocampus (93). Further investigation is needed to explore the mechanisms associated with other metabolites.

### 3.2 Neurotransmitters

#### 3.2.1 Monoamine neurotransmitters

Monoamine neurotransmitters, including catecholamines such as DA, NE, and epinephrine, as well as indoleamines like 5-HT, play a crucial role in mood regulation and are closely associated with depression (94). Current clinical antidepressants primarily rely on the “amine depletion hypothesis,” which involves inhibiting enzymes related to monoamine metabolism to increase synaptic levels of 5-HT, NE, and DA, thereby achieving therapeutic effects (95). The gut microbiota is essential in modulating monoamine neurotransmitter levels, which may influence the onset of depression (83, 96).

##### 3.2.1.1 NE

NE and the norepinephrine transporter (NET) are vital in the pathophysiology of depression. NE binds to NET to recycle excess NE in the synaptic cleft and maintain NE metabolic homeostasis. In the thalamus, increased NET availability accelerates NE recycling in the synaptic cleft, reducing NE signaling and leading to attention deficits (97). The locus coeruleus (LC) is the main source of NE in the brain (98). In depressed patients, reduced NET concentration in the LC results in NE accumulation in the synaptic cleft, disrupting NE signaling and impairing emotional regulation and cognitive function (99).

α2-Adrenergic receptors function as auto-receptors for NE neurons, regulating NE release through negative feedback. In depression patients, increased binding of α2-adrenergic receptors to agonist ligands in NE neuron cytoplasm enhances NE autoreceptor activity, diminishing noradrenergic neurotransmission (100). α-Adrenergic receptors are significant in depression, and antagonizing α2-adrenergic receptors may be a potential therapeutic approach, although direct evidence is limited (101).

Moreover, research indicates that β-adrenergic receptors may enhance stress resistance, offering new insights for depression treatment (102). Chronic antidepressant use affects NE system function. Antidepressants may reduce both the quantity and functionality of β-adrenergic receptors by lowering the sensitivity of NE-responsive adenylate cyclase enzymes, with 5-HT also playing a role. Studies show that 5-HT, which inhibits NE release, interacts with NE, which in turn reduces serotonergic neuron activity (103–105). This interaction is crucial for addressing treatment-resistant depression. Current depression therapies rely on NE reuptake inhibitors and 5-HT reuptake inhibitors, with the interplay between 5-HT and NE potentially impacting therapeutic efficacy and guiding clinical therapy selection (106). Clinically, combining SSRIs with agonists that enhance NE release has shown efficacy in treatment-resistant cases (107). While NE system dysregulation is established in depression, key gaps remain in understanding receptor subtype-specific contributions and optimal therapeutic modulation. The development of CNS-penetrant adrenergic receptor modulators with improved selectivity could address current limitations in targeting noradrenergic pathways. Further elucidation of 5-HT/NE interactions may reveal novel strategies for treatment-resistant cases, particularly through combined receptor modulation approaches. Clinical translation would benefit from advanced NET imaging techniques to guide personalized treatment selection and monitor therapeutic response.

##### 3.2.1.2 5-HT

The primary site of 5-HT production is the intestinal enterochromaffin (EC) cells. These cells absorb tryptophan from the diet via the bloodstream and then convert it into 5-HT through the action of tryptophan hydroxylase (108). This process is governed by gut microbiota, including *Lactobacillus* and *Bifidobacterium*, which enhance 5-HT synthesis by modulating tryptophan metabolism and augmenting tryptophan availability in the gut (109, 110). 5-HT plays a crucial role in modulating macrophage polarization by upregulating the expression of genes associated with M2-type macrophages, such as SERPINB2 and THBS1, while simultaneously downregulating the expression of genes characteristic of M1-type macrophages. This dual action effectively reduces the inflammatory response (111). Furthermore, 5-HT regulates immunological responses and systemic signaling through the vagus nerve, consequently influencing gastrointestinal and central nervous system activities. In patients with depression, the concentration of 5-HT and the activity of the 5-HT transporter are reduced, particularly in brain areas such as the amygdala and striatum. This reduction leads to impaired 5-HT signaling, which in turn increases the likelihood of developing depression (112, 113).

##### 3.2.1.3 DA

Roughly 90% of human DA is produced in the gut by *Bacillus* and *Serratia marcescens* (114, 115). Most of the L-DOPA synthesized in the gut is transported across the BBB to the brain, where it is subsequently converted into DA. The DA function is influenced by multiple factors. Folate deficiency disrupts homocysteine metabolism, while increased homocysteine levels cause neurotoxicity and damage dopaminergic neurons (83). T-2 toxin, an environmental contaminant generated from fungi, can elicit depressive-like behaviors in mice by increasing DA transporter levels in the nucleus, thereby impairing DA metabolism (116). Furthermore, age-related pro-inflammatory mechanisms correlate with a reduction in DA signaling efficacy, indicating that older adults may represent a lager proportion of individuals with depression (117). Dopaminergic signaling directly influences mood, and its dysfunction is closely associated with depression. Specifically, symptoms of depression such as anhedonia are related to the dysregulation of dopamine signaling (118). Research indicates that DA agonists are useful in alleviating depression linked to Parkinson’s disease; however, there is less empirical data about their efficacy in treating depression more broadly (119).

#### 3.2.2 GABA

Gut microbiota, including *Corynebacterium glutamicum*, *Lactobacillus plantarum*, and *Lactococcus lactis*, produce GABA through a metabolic pathway. This process involves the isomerization of L-glutamic acid to D-glutamic acid, which is then decarboxylated to form GABA (120). Due to the limited dietary GABA content, the current emphasis is on enhancing *in vivo* levels by manipulating the population of GABA-producing microorganisms (121). GABA crosses the intestinal barrier and binds to receptors on enteric neurons and the vagus nerve, thereby regulating intestinal function. It penetrates the central nervous system through specific transporters at the BBB, binding to GABA receptors on post-synaptic neurons to inhibit the influx of Na^+^, K^+^, Ca2^+^, and Cl^−^, consequently diminishing neuronal excitability and facilitating various physiological functions, including the promotion of sleep and the alleviation of anxiety (122–124). Subsequent research indicates that chronic stress suppresses the GABAergic neural network, resulting in depression-like behavior, which can be ameliorated with GABA supplementation or probiotic intervention (121, 125). Specific processes involve (1) Administration of *Lactobacillus rhamnosus JB-1* modulates the activity of GABA receptors (GABARs) in the brain, thereby resulting in decreased anxiety and depression (23). (2) Gut microbiota metabolites, including SCFAs, are able to permeate the BBB and participate in the GABA-glial cell cycle to mitigate depression-like symptoms (126). Nonetheless, current intervention techniques exhibit dose-effect variability and mechanistic intricacy, requiring additional elucidation of strain specificity and action targets.

#### 3.2.3 Neuropeptides

Neuropeptides are produced by neuroglial cells inside the enteric nervous system and can convey messages to remote organs (83). Alterations in neuropeptide concentrations have been noted in neurological illnesses linked to intestinal inflammation, indicating that immune-neurotransmitter interactions play a role in neurogenesis. Neuropeptide levels have been correlated with depression (83). The precise mechanisms of neuropeptide antidepressants are as follows: Stimulation of the GLP-1 receptor inhibits the synthesis and secretion of corticotropin-releasing hormone (CRH). This mechanism effectively diminishes the overactivation of the HPA axis and attenuates brain damage resulting from chronic stress. Additionally, the engagement of peptide YY and GLP-1 with receptors in the vagal nerve terminals, such as the GLP-1 receptor and neuropeptide Y receptor Y2, transmits signals via vagal afferent fibers to the nucleus tractus solitarius (NTS) in the brainstem. Subsequently, the NTS transmits these impulses to the hypothalamic paraventricular nucleus, suppressing CRH neuronal activity. This interaction facilitates monoamine neurotransmitters’ release, thereby enhancing mood. The signal may also be conveyed to the prefrontal cortex, hippocampus, and other areas to augment neuroplasticity (e.g., BDNF expression) and ameliorate neuronal damage linked to depression (16, 127). Recent studies have found that the tetrapeptide N-acetyl-serine-aspartyl-lysyl-proline (Ac-SDKP) can inhibit neuroinflammatory signaling and alleviate depressive-like behavior in mice (128). Auxin-releasing peptides are closely related to mental disorders and can alleviate depression by activating the HPA axis and promoting DA release (129, 130). Measuring growth hormone-releasing peptide expression levels can also distinguish between depression and bipolar disorder, with the latter having higher growth hormone-releasing peptide levels (119). Targeted growth hormone-releasing peptide therapy for neuropsychiatric disorders is a highly promising and rapidly evolving approach. Recent clinical studies have reported a significant association between elevated growth hormone-releasing peptide levels and reduced depressive symptoms in patients with depression treated with probiotics. However, the exact efficacy of this therapy for depression remains uncertain. Therefore, further clinical trials should be conducted to investigate the efficacy of this growth hormone-releasing peptide (131).

### 3.3 Nervous pathways

#### 3.3.1 Vagus nerve

The vagus nerve is integral to the MGB axis and functions as the principal conduit for neuronal communication between the gut and the brain (132). The vagus nerve is crucial in neurological and mental disorders (133). The gut microbiota affects intestinal luminal metabolites, which stimulate chemosensory vagal afferent fibers to relay signals to the brain (134). In 2005, vagus nerve stimulation (VNS) has been approved for use in treating treatment-resistant depression, yet its invasiveness has rendered it difficult for many patients to accept. Researchers have been investigating noninvasive alternative therapies (135). Recently, transcutaneous vagus nerve stimulation (tVNS) has enabled noninvasive therapeutic approaches (136). tVNS activates the cortex through electrically modulating the vagus nerve’s auricular branch, thereby stimulating the subcortical nuclei. tVNS is effective in improving depressive symptoms (137, 138). The specific mechanisms include: tVNS augmenting the passage of monoamine neurotransmitters across synapses, rectifying neurochemical and physiological irregularities linked to depression, and improving cognitive performance (139). It facilitates the production of acetylcholine, which attaches to cholinergic receptors on immune cells, suppresses the release of inflammatory mediators, and produces an antidepressant effect (140). tVNS, as a crucial adjunctive treatment for patients with refractory depression, markedly alleviates depressed symptoms when administered with traditional antidepressants, and is anticipated to emerge as a vital supplementary instrument in future depression management (141).

#### 3.3.2 Enteric nervous system

Enteric nervous system (ENS) is an autonomous neuronal plexus within the digestive tract, commonly termed “second brain.” The development initiates with the proliferation and migration of embryonic enteric neural crest cells, governed by the glial cell-derived neurotrophic factor/growth factor receptor signaling pathway, and advances in reaction to gut microbiota colonization (142, 143). ENS is composed of the myenteric plexus, which modulates gut motility, and the submucosal plexus, which controls intestinal secretion.

ENS has a dual purpose. It can autonomously modulate intestinal physiological functions and also lead to depression’s etiology. Enteric glial cells (EGCs) and enteroendocrine cells (EECs) are integral to these processes and are significantly linked to the onset of depression. Research clarifies the interaction between other MGB axis pathways and the enteric nervous system in mediating depression. Germ-free mice demonstrate diminished synaptic growth and a lower density of intermuscular neurons, suggesting that gut microbiota is important in the formation of enteric neurons (143); SCFAs maintain ENS function by restoring neuronal loss (144); reelin, an intrinsic extracellular matrix protein, amalgamates immunomodulatory and neuroplasticity roles within the ENS while upholding intestinal barrier integrity, potentially affecting depression (145, 146); chronic stress activates the HPA axis, leading to an inflammatory subset of EGCs that initiate intestinal inflammation through the CSF1/TNF-α pathway, hence leading to depression (147, 148). EGC is essential for preserving the intestinal epithelial barrier. The ECC releases neuropeptides, including cholecystokinin, transmitting signs from epithelium to submucosal plexus and central nervous system, therefore modulating metabolism (e.g., 5-HT and kynurenine) within the MGB axis (149, 150). Moreover, LPS stimulation in mice enhances gut microbiota growth and repairs damaged neurons without generating new neurons, further emphasizing the interconnectedness and inseparability of MGB axis metabolic pathways (144).

### 3.4 Cell signaling pathways

Depression is intricately linked to disrupted cellular signaling networks (83). The gut microbiota modulates interactions along the MGB axis via the following fundamental pathways: (1) MAPK/CREB pathway: Gut microbiota activates MAPK pathway via regulation of post-translational changes, resulting in the induction of depression-like behaviors. The downstream CAMK II-CREB cascade is a crucial modulator of signaling within the gut microbiota-MGB axis (151–153). (2) Endogenous cannabinoid system: The endogenous cannabinoid system regulates memory-emotion encoding by the binding of endogenous cannabinoids to hippocampus-specific receptors, which is directly associated with the depressive phenotype (83, 154). (3) mTOR pathway: The deregulation of the mTOR pathway results in compromised synaptic plasticity and neurobehavioral anomalies, which are strongly linked to mood disorders (155). (4) Heat shock protein pathway: Heat shock proteins contribute to the etiology of depression by modulating BDNF expression, preserving neuroprotection and cognitive function, and affecting the HT22 hippocampal cell line and hippocampus tissue (156).

### 3.5 Immunity

The immune response serves as a crucial pathway through which the gut microbiome affects depression. (1) Centralized migration and modulation of immune cell subsets. Initially, gut microbiota-activated natural killer (NK) cells traverse to the central nervous system (CNS), promoting anti-inflammatory astrocytes’ formation and mitigating neuroinflammation by triggering T-cell death through the TRAIL-DR5 signaling pathway, thus providing a protective effect against depression (157). Secondly, IgA^+^ plasma cells originating from the gastrointestinal tract traverse to the meninges through the lymphatic system, and their deficiency leads to diminished central resistance to infection in germ-free animals (158). The gut microbiota affects intrinsic lymphocytes to modulate antigen presentation mechanisms, creating a robust connection with the host’s immune system (159). Fourth, Treg cells are diminished in individuals with mood disorders, but *Clostridium difficile* can supply growth factors like TGF-β to intestinal Treg cells, thus modulating intestinal immune homeostasis and aiding in mood preservation. Fifth, T cell activation in depressed individuals and the downregulation of genes associated with humoral immunity in chronic unpredictable mild stress-induced sad mice indicate that both innate and adaptive immunity lead to depression’s etiology. Interaction between gut bacteria and immune cells can both facilitate immunological tolerance and induce inflammation, thereby serving a dual role in regulating immunity (160–162). (2) Inflammatory factor levels are heightened, while anti-inflammatory factor levels are diminished in individuals with depression (163). Initially, concentrations of the anti-inflammatory cytokine IL-22 are markedly diminished in individuals with depression, and the administration of exogenous IL-22 ameliorates stress-induced depressive behaviors (164, 165). Secondly, IL-10 was markedly enhanced in the plasma of patients treated with *Lactobacillus rhamnosus*, indicating a widespread neuroimmune influence, although no behavioral effects have been documented (166). Third, the breakdown of the intestinal barrier activates the LPS/TLR4 pathway, resulting in endotoxin translocation and markedly increased serum anti-LPS IgM/IgA concentrations in patients (167, 168). Fourth, pro-inflammatory variables cause an imbalance in kynurenine metabolism, hence enhancing the formation of the neurotoxic compound quinolinic acid and inducing neurotoxicity. (3) Immunometabolism of glial cells. Inflammatory responses may impair neuroplasticity and provoke neuroinflammation. Microglia activation results in the excessive secretion of pro-inflammatory chemicals, which disrupt neuronal connectivity (169, 170). Furthermore, microglial dysfunction impedes hippocampus formation and intensifies depression symptoms (171). Astrocytes modulate microglial activity by preserving the neurochemical milieu and overseeing the BBB, whereas systemic inflammatory reactions induce astrocytic atrophy, worsen microglial dysfunction, and intensify neuroinflammation (172). Dysbiosis additionally prompts excessive generation of reactive oxygen species and impaired mitochondrial autophagy, worsening the crisis in neuronal energy metabolism (173). Recent investigations have demonstrated that the immune system engages with additional channels of the MGB axis. The iPSC-immune cell co-culture paradigm demonstrates that colony metabolites, such as SCFAs, modulate Treg/Th17 homeostasis via epigenetic reprogramming through HDAC inhibition, thereby presenting a novel approach for understanding immune-neurological interactions (174). The gut microbiome influences depression through immune pathways, but key questions remain about how specific microbes drive neuroprotective versus inflammatory responses in humans. Future research should clarify these mechanisms using human cell models and identify immune biomarkers to guide personalized probiotic therapies for depression.

### 3.6 HPA axis

As a crucial neuroendocrine regulatory axis in the human endocrine system, the HPA axis is the core system regulating the stress response (175). When the body perceives a stress stimulus, the hypothalamus secretes CRH, thereby activating the HPA axis, which subsequently stimulates the pituitary gland to secrete adrenocorticotropic hormone (ACTH) and ultimately leads to the release of cortisol by the adrenal glands (176). The gut microbiota can influence HPA axis activation by regulating stress hormone levels, including cortisol (177). Studies have demonstrated that ACTH levels are significantly elevated in germ-free mice but return to normal levels following the transplantation of normal feces (177). Furthermore, studies have found that the gut microbiota can regulate the circadian secretion of cortisol, and depletion of the gut microbiota can lead to disruption of the glucocorticoid circadian rhythm (178). This finding was further confirmed by gut microbiota transplantation, with *Lactobacillus reuteri* identified as the key bacterial species involved in this process (178).

It has been demonstrated that the HPA axis is dysregulated in patients with depression, with its overactivation leading to chronic stress responses (179). Overactivation of the HPA axis leads to the release of large amounts of glucocorticoids. In germ-free mice, glucocorticoid receptors are overexpressed and functionally enhanced, leading to impaired immune function, which may be associated with the onset of depression (159). However, a study has shown that HPA activation can induce depression-like behavior by disrupting glucocorticoid receptor expression in the hippocampus (180). Therefore, the specific role of glucocorticoid receptors in HPA axis-mediated depression onset requires further investigation. In addition to its involvement in depression onset through glucocorticoids and their receptors, HPA axis activation can increase intestinal permeability, leading to bacterial translocation and exacerbating inflammatory responses (162). Inflammatory responses are closely associated with the onset and progression of depression. Additionally, in rodent models, vagus nerve stimulation increases CRF mRNA expression in the hypothalamus and elevates plasma ACTH and corticosterone levels (161). This suggests a close interaction between the vagus nerve and the HPA axis, thereby influencing the onset of depression.

Current research indicates that the HPA axis not only serves as a biomarker for the onset of depression but that changes in related hormone levels, such as elevated cortisol levels in the morning and at night, are considered risk factors for the onset of depression (181, 182). For example, fluctuations in ovarian hormones and neurosteroids during perimenopause can alter GABA’s regulation of the HPA axis, leading to HPA axis dysfunction and increased vulnerability to depression (183). Additionally, genetic variations and activity of the HPA axis are considered important predictors of depression and cognitive function in patients. For example, glucocorticoid receptor genetic variations are associated with attention and working memory, while mineralocorticoid receptor is associated with language memory (184).

### 3.7 Non-bacterial gut microbes influence depression through the MGB axis

**Figure 4:**
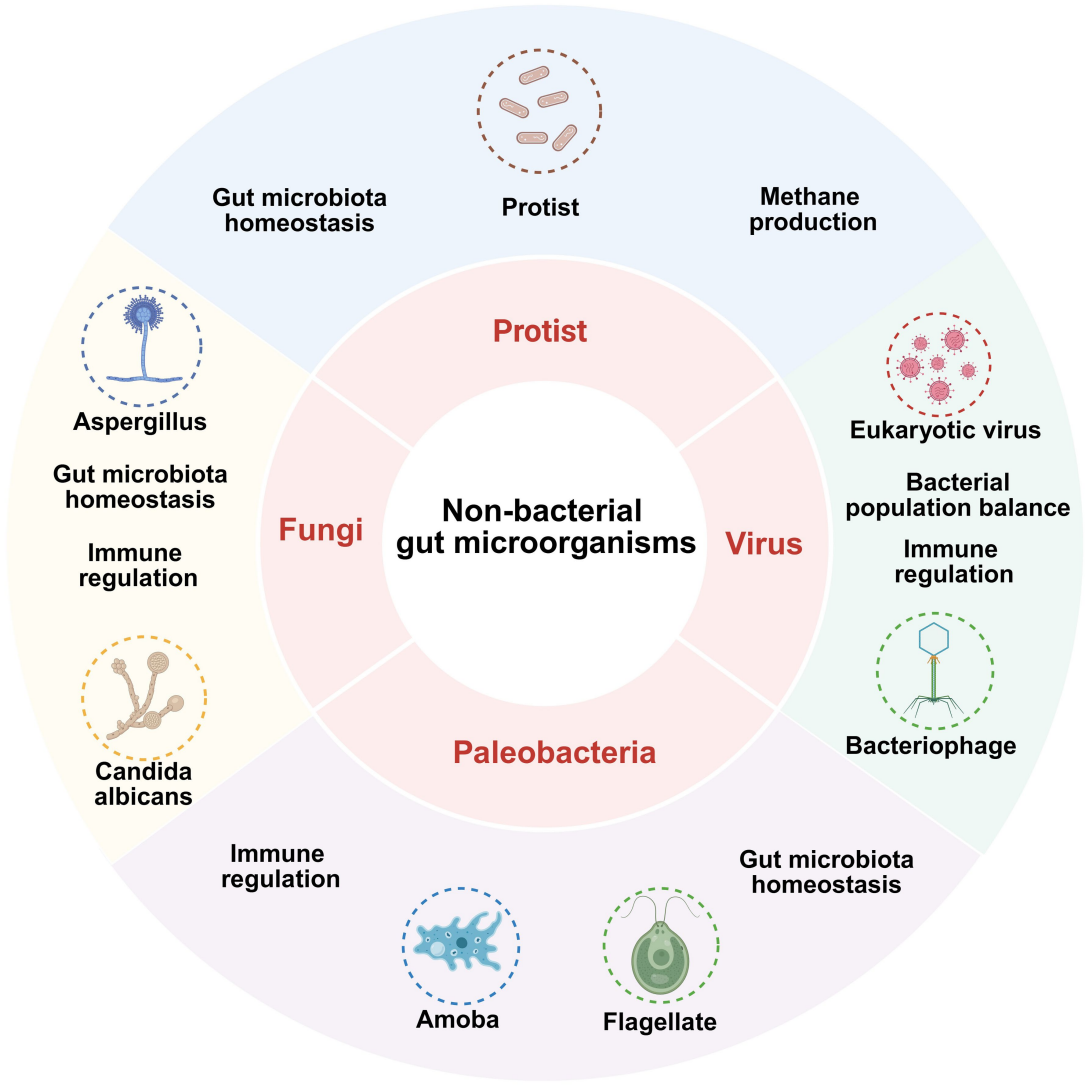
Mechanisms by which nonbacterial gut microbiota affect depression.

Although fungi, viruses, and phages form a relatively small part of the gut microbiota, their significance cannot be overlooked and is now receiving more attention (Figure 4).

Fungi such as *Aspergillus* and *Candida albicans* contribute to the maintenance of immune regulation and gut microbiota homeostasis; protists are involved in methanogenesis and gut microbiota homeostasis; viruses such as eukaryotic virus affect gut health by regulating bacterial population balance and immune responses; paleobacteria such as *Amoeba* and *Flagellate* are also involved in immune regulation and maintenance of gut homeostasis.

*Pichia pastoris KM71H* and *Saccharomyces boulardii* demonstrate antidepressant effects via immunomodulation, restoration of the intestinal and BBB, antioxidative stress mitigation, modulation of intestinal microbiota composition, reduction of corticosterone levels in the hippocampus of mice, and inhibition of HPA axis hyperactivation; they are classified as intestinal probiotic fungi (160, 166, 174).

β-glucan (BG) produced by *Saccharomyces cerevisiae* may influence depression via twin mechanisms: (1) gut microecological regulation: BG enhances the prevalence of advantageous gut bacteria and elevates the concentration of SCFAs, while also restoring the intestinal barrier. (2) Control of neuroinflammation: reducing microglial activation within the hippocampal region and blocking the IL-6-driven pathway of astrocyte apoptosis (185, 186). BG has demonstrated the potential to alleviate symptoms of Alzheimer’s disease, while its direct effectiveness in treating depression necessitates additional elucidation (186).

The known association between human immunodeficiency virus and hepatitis C virus and the risk of depression is acknowledged; nevertheless, the mechanism by which enteroviruses (EV) influence depression via the MGB axis remains contentious (187, 188). Research involving animals has shown that Coxsackievirus B-3 infection inhibits both humoral and cellular immunity, and Coxsackievirus infection in children correlates with heightened anxiety symptoms, although enterovirus infection in children does not elevate the risk of depression (189–191).

## 4 The impact of gut microbiota on bipolar disorder via the gut-brain axis

Bipolar disorder (BD), also known as manic-depressive disorder, is a common mental disorder characterized by both manic episodes and depressive episodes (a typical feature), differing from depression (192). Gut microbiota is significantly associated with bipolar disorder. Patients with BD exhibit significantly reduced α diversity in gut microbiota, increased abundance of the *genera Atopobium*, *Bacteroidetes*, *Escherichia-Shigella*, and *Flavonifractor*, and reduced abundance of the *phylum Firmicutes* (21, 193, 194). *Faecalibacterium* is negatively correlated with depression severity (195). The mechanism by which the gut-brain axis functions in bipolar disorder is broadly similar to its role in depression. The *genus Oscillospira* can inhibit the progression of bipolar disorder by producing anti-inflammatory metabolites such as butyrate and participating in fiber fermentation mechanisms, and it is negatively correlated with bipolar disorder (33, 196, 197). B vitamins also play a role in the pathogenesis of BD (198). In the tryptophan metabolic pathway, reduced abundance of KO0837 (aromatic amino acid transferase) and K01667 (tryptophanase) may lead to reduced tryptophan synthesis, while increased abundance of genes such as K00626 (acetyl-CoA transferase) may affect mitochondrial function and neurotransmitter balance. Tryptophan is a precursor to serotonin and kynurenine, and its metabolic pathway undergoes significant changes in BD patients (199). Additionally, studies have found that brain structure acts as an intermediary factor in the influence of gut microbiota on mental disorders, with 13 complete mediation effects identified in BD, including left ventral hypothalamic volume and cerebellar peduncle pathways (196). Bipolar disorder severely impacts patients’ mental health, daily functioning, and quality of life, and is a chronic, recurrent condition. The therapeutic potential of the gut microbiota in mental and psychological disorders is increasingly attracting attention from scientists. Currently, most studies on the gut microbiota and bipolar disorder are conducted in conjunction with other mental disorders, necessitating further independent research on this topic. In the future, combining multi-omics data (such as metabolomics and proteomics) with longitudinal studies could further elucidate the specific mechanisms of the gut-brain axis, providing new insights for the treatment of bipolar disorder.

## 5 Discussion

Depression, a critical worldwide health issue with profound effects on human well-being and economic status, necessitates heightened scientific focus (200). Recently, the significance of gut microbiota concerning depression has emerged as a prominent area of research. The MGB axis functions as a two-way communication pathway between brain and gut, significantly advancing the research of its involvement in depression (16). This paper comprehensively investigates the mechanisms by which gut microbiota influence depression through MGB axis, systematically summarizes the roles of SCFAs metabolism, tryptophan metabolism, and monoamine neurotransmitters in depression’s pathogenesis, and emphasizes recent research advancements, including the potential roles of sphingolipid metabolism, cellular signaling pathways, and neuropeptides in the etiology of depression, to propose novel strategies for its diagnosis and treatment.

Nonetheless, it is crucial to acknowledge that current investigations possess limits and numerous unresolved enquiries persist, necessitating additional experimental confirmation. Initially, variations in the same microbiota species may differ among depressed individuals of diverse ages and genders, hence precluding the utilization of specific alterations in microbiota as a singular predictor of the onset or advancement of depression (27). Furthermore, despite the acknowledged heterogeneity in individual gut microbiota composition, this variability must be taken into account when targeting gut microorganisms for depression’s treatment. Precise assessment of individual gut microbiota composition has recently been suggested via the integrated study of metagenomics and metabolomics; nevertheless, further studies are required to validate its efficacy. The intricate nature of human gut microbiota, which cannot be entirely replicated by cellular and animal models, renders the translation of fundamental research findings into clinical applications difficult. Current clinical investigations are predominantly observational and are unable to determine a causal association between gut microbiota dysbiosis and depression. Future intervention studies, including probiotic therapy or fecal microbiota transplantation, are necessary to assess enhancements in depressive symptoms and so validate the involvement of gut microorganisms in depression (201, 202). Concurrently, longitudinal cohort studies employing Mendelian randomization analyses are essential to eliminate the influence of environmental factors, including nutrition and stress.

Besides the indirect role of gut microbes in depression through the MGB axis, damage to the BBB in depressed individuals facilitates gut microbiota and their metabolites’ translocation to the brain, resulting in spatially and temporally specific neuroinflammation (31); however, this phenomenon has not been conclusively validated by experimental evidence. Future research may figure out the potential impairment of BBB integrity in individuals with depression, the role of gut microbiota in exacerbating neuroinflammation through this mechanism, and strategies for intervening in the course of depression by focusing on BBB protection. The process via which gut bacterial metabolites penetrate the brain through the intestinal barrier as opposed to crossing the BBB to induce neuroinflammation remains inadequately elucidated. Recent investigations utilizing an iPSC model have elucidated the metabolite cross-barrier process. A co-culture system of induced pluripotent stem cell-derived intestinal epithelial cells (iIECs) and intestinal brain microvascular endothelial cells (iBMECs) demonstrated that butyric acid traverses the BBB through the MCT and stimulates hippocampal BDNF production (93). Additional research is required to validate the processes of other metabolites. Moreover, while SCFAs are known to enhance intestinal barrier integrity, current research indicates that elevated levels of SCFAs may compromise the intestinal barrier (40). Additional validation of the effects of varying SCFA concentrations on intestinal barrier integrity and their influence on the efficacy of depression treatment is required. Simultaneously, the incidence of depression rises among postmenopausal women, potentially linked to estrogen levels, resulting in the suggestion of the “hormone-gut microbe-depression axis” (203). Additional investigation is required to examine this concept. Moreover, the temporal aspects of inflammation in the brains of individuals with depression remain inadequately substantiated. If this hypothesis holds, might the identification of inflammatory markers in the brain or imaging findings establish a foundation for temporal depression interventions, such as anti-inflammatory therapies during the acute phase and anti-neurodegenerative treatments in the chronic phase (31)? METH-exposed mice demonstrated depressive-like behavior; nevertheless, their gut microbiota composition remained mostly unchanged (92). Nevertheless, antibiotic therapy mitigated increased serum TMAO concentrations and depressive-like behaviors (92). The correlation between TMAO and depression, as well as its underlying mechanisms, warrants thorough examination. Although the role of α-adrenergic receptors in depression have been described, direct evidence is still limited. Antagonizing α2-adrenergic receptors may be a promising strategy for treating depression, which requires further experimental verification (101). The role of gut microorganisms in the MGB axis, which frequently leads to comorbidity between depression and other illnesses like anorexia nervosa, necessitates additional investigation (204, 206). Currently, in addition to emerging advances in the treatment of depression based on gut microbiota and the gut-brain axis, treatment methods and approaches for depression are also constantly evolving. Ketamine, dextromethorphan, brain-computer interface technology, and photobiomodulation methods have all shown potential therapeutic effects for depression. In the future, the treatment of depression will undoubtedly become more diversified, with the use of multiple drugs in combination to enhance efficacy. In summary, while substantial advancements have been achieved in examining the correlation between gut bacteria and depression, multiple foundational investigations have elucidated the gut microbes-MGB axis-depression route. Beneficial gut bacteria have been utilized in clinical research to modulate depression, demonstrating modest benefit. Nevertheless, other topics require additional investigation. Future research should prioritize experimental validation and individual variability to enhance strategies for the diagnosis and treatment of depression.
